# Supplementary material for: Experience, Knowledge, and Perceptions of Pharmacogenomics among Pharmacists and Nurse Practitioners in Alberta Hospitals
Source: Pharmacy (Basel). 2022 Oct 26;10(6):139. doi: 10.3390/pharmacy10060139 (PMC9680290; doi:10.3390/pharmacy10060139)
Supplement: Supplementary file 1 [file pharmacy-10-00139-s001.zip › SUPPLEMENT-SURVEY.pdf]

Experience, Knowledge, and Perceptions of Pharmacogenomics among Healthcare Professionals in Alberta Hospitals

SUPPLEMENTARY MATERIALS

Survey Questions

Eligibility Questions

Before beginning the survey please answer the following three questions to determine if you are eligible to participate in this study.

1. Do you currently practice within an Alberta Health Services facility/setting?
   - ☐ Yes
   - ☐ No [triggers stop survey]

2. What is your current profession within Alberta Health Services?
   - ☐ Physician
   - ☐ Pharmacist
   - ☐ Nurse Practitioner
   - ☐ None of these options [triggers stop survey]

3. Do you provide direct patient care in your current role?
   - ☐ Yes
   - ☐ No [triggers stop survey]

Part 1A: Demographics / Professional History

The following 4 questions will gather information about your current professional environment. You may decline to answer any questions within this portion of the survey by selecting "prefer not to say". Survey responses will only be presented in aggregate, with possible further collation of responses, to maintain anonymity.

1. How many years have you been practicing <u>in the profession you identified at the beginning of this survey</u>?
   - ☐ Less than 2 years
   - ☐ 2-5 years
   - ☐ 6-10 years
   - ☐ 11-15 years
   - ☐ 16-20 years
   - ☐ More than 20 years
   - ☐ Prefer not to say
2. What size of community do you currently practice in?
   - ☐ Locum (varied community sizes)
   - ☐ Less than 1,000 inhabitants
   - ☐ 1,001-10,000 inhabitants
   - ☐ 10,001-50,000 inhabitants
   - ☐ 50,001-100,000 inhabitants
   - ☐ 100,001-250,000 inhabitants

- ☐ Greater than 250,000 inhabitants
- ☐ Prefer not to say

3. What clinical area do you specialize in?
    - ☐ No specialty/ general practice
    - ☐ Emergency
    - ☐ Cardiovascular or Stroke
    - ☐ Psychiatry
    - ☐ Gastroenterology
    - ☐ Infectious Diseases
    - ☐ Oncology
    - ☐ Respiratory
    - ☐ Nephrology
    - ☐ Endocrinology or Diabetes
    - ☐ Geriatrics
    - ☐ Pediatrics
    - ☐ Critical Care or ICU
    - ☐ Palliative Care
    - ☐ Other (please specify clinical area: ___________________)
    - ☐ Prefer not to say
4. What is your primary setting within Alberta Health Services
    - ☐ Inpatient
    - ☐ Outpatient/ambulatory
    - ☐ Both inpatient and outpatient
    - ☐ Prefer not to say

Part 1B: Pharmacogenomics Background

The following questions will gather information about your prior education and experience in pharmacogenomics.

For the purposes of this survey, pharmacogenomics refers to the study of how a person's genome influences their response to medications.

1. Have you received any prior education or training in pharmacogenomics?
    - ☐ Yes
    - ☐ No

    1.1. [Branching logic, question appears if "Yes" selected to the previous question] Have you received prior education in pharmacogenomics during… (check all that apply):
    - ☐ Your qualifying professional degree program
    - ☐ Other degree programs (pre or post-professional)
    - ☐ A conference, congress, or meeting
    - ☐ On-site training in a professional practice environment (excluding during qualifying degree program)
    - ☐ A certificate program
    - ☐ Self-study
    - ☐ Other (please specify where you have learned about pharmacogenomics before:_)
    - ☐ Prefer not to say
2. Have you previously applied pharmacogenomics in practice?
    - ☐ Yes
    - ☐ No

[Branching logic, questions 2.1-2.3 appear if “Yes” selected to the previous question]

2.1. How have you applied pharmacogenomics in practice? (check all that apply)
- ☐ Provided pre-test education/obtained consent for testing
- ☐ Ordered pharmacogenomic test
- ☐ Interpreted pharmacogenomic test results (genotype to phenotype)
- ☐ Formulated medication therapy plan based on pharmacogenomic test results
- ☐ Provided patient education for pharmacogenomic test results or resultant medication therapy plan
- ☐ Provided other healthcare provider education on pharmacogenomic test results or medication therapy plan
- ☐ Other (please specify how you have applied pharmacogenomics in practice: ___)
- ☐ Prefer not to say

2.2. In your career, how many patients have you recommended, ordered, assessed, or otherwise utilized pharmacogenomic test results for? (i.e. how many patients apply to the responses provided in the last two questions?)
- ☐ None (prior experience in pharmacogenomics was not in direct patient care)
- ☐ <10
- ☐ 10-50
- ☐ 51-100
- ☐ >100

2.3. Which pharmacogenetic testing providers have conducted testing for your patients (select all that apply)
- ☐ Assurex/Myriad (GeneSight)
- ☐ BiogeniQ
- ☐ CEN4GEN
- ☐ Color Genomics
- ☐ DNALabs (MatchMyMeds)
- ☐ Dynacare/Genomind (Genecept)
- ☐ GeneYouIn (Pillcheck)
- ☐ GenXys/LifeLabs (TreatGx)
- ☐ Inagene
- ☐ MyDNA
- ☐ Neuropharamgen
- ☐ OneOme (RightMed)
- ☐ Personalized Prescribing (RxReport)
- ☐ PurePharm
- ☐ Unsure / Do not know
- ☐ Other (please specify pharmacogenetic testing company/lab used: __________)

3. Please indicate your level of knowledge and familiarity regarding the following pharmacogenomics resources/guidelines:

3.1. Pharmacogenomics Knowledge Base (PharmGKB) website
- ☐ I have never heard of this resource
- ☐ I have heard of, but am not familiar with this resource
- ☐ I have heard of, am familiar with, but have never used this resource in evaluating PGx information
- ☐ I have heard of, am familiar with, and have used this resource to evaluate PGx information

3.2. Clinical Pharmacogenetics Implementation Consortium (CPIC®) website and guidelines

- ☐ I have never heard of this resource
- ☐ I have heard of, but am not familiar with this resource
- ☐ I have heard of, am familiar with, but have never used this resource in evaluating PGx information
- ☐ I have heard of, am familiar with, and have used this resource to evaluate PGx information

3.3. Dutch Pharmacogenetics Working Group (DPWG) Recommendations
- ☐ I have never heard of this resource
- ☐ I have heard of, but am not familiar with this resource
- ☐ I have heard of, am familiar with, but have never used this resource in evaluating PGx information
- ☐ I have heard of, am familiar with, and have used this resource to evaluate PGx information

3.4. Canadian Pharmacogenomics Network for Drug Safety (CPNDS) Website and Guidelines
- ☐ I have never heard of this resource
- ☐ I have heard of, but am not familiar with this resource
- ☐ I have heard of, am familiar with, but have never used this resource in evaluating PGx information
- ☐ I have heard of, am familiar with, and have used this resource to evaluate PGx information

3.5. Pharmacogene Variation Consortium (PharmVar) website / data repository
- ☐ I have never heard of this resource
- ☐ I have heard of, but am not familiar with this resource
- ☐ I have heard of, am familiar with, but have never used this resource in evaluating PGx information
- ☐ I have heard of, am familiar with, and have used this resource to evaluate PGx information

3.6. Please list any other pharmacogenomics resources you are familiar with. ________

4. Has a patient ever asked you about pharmacogenetic testing?
   - ☐ Yes
   - ☐ No
5. Have you seen/assessed a patient with pharmacogenetic test results that you did not order?
   - ☐ Yes
   - ☐ No

Part 2: Confidence & Self-Rated Knowledge

The following 11 questions evaluate your confidence and self-rated knowledge of pharmacogenomics.

1. I understand the basic concepts of genetics
   - ☐ Strongly Disagree
   - ☐ Disagree
   - ☐ Neutral
   - ☐ Agree
   - ☐ Strongly Agree
   - ☐ Prefer not to say
2. I understand the basic concepts of pharmacogenomics/pharmacogenetics
   - ☐ Strongly Disagree

- ☐ Disagree
- ☐ Neutral
- ☐ Agree
- ☐ Strongly Agree
- ☐ Prefer not to say

3. I can identify patients suitable for pharmacogenomic testing
   - ☐ Strongly Disagree
   - ☐ Disagree
   - ☐ Neutral
   - ☐ Agree
   - ☐ Strongly Agree
   - ☐ Prefer not to say
4. I can identify medications suitable for pharmacogenomic testing
   - ☐ Strongly Disagree
   - ☐ Disagree
   - ☐ Neutral
   - ☐ Agree
   - ☐ Strongly Agree
   - ☐ Prefer not to say
5. I can identify an appropriate laboratory to perform pharmacogenomic testing
   - ☐ Strongly Disagree
   - ☐ Disagree
   - ☐ Neutral
   - ☐ Agree
   - ☐ Strongly Agree
   - ☐ Prefer not to say
6. I am aware of the risks of pharmacogenomic testing
   - ☐ Strongly Disagree
   - ☐ Disagree
   - ☐ Neutral
   - ☐ Agree
   - ☐ Strongly Agree
   - ☐ Prefer not to say
7. I can communicate the risks of pharmacogenomic testing
   - ☐ Strongly Disagree
   - ☐ Disagree
   - ☐ Neutral
   - ☐ Agree
   - ☐ Strongly Agree
   - ☐ Prefer not to say
8. I can translate a genotype into a phenotype
   - ☐ Strongly Disagree
   - ☐ Disagree
   - ☐ Neutral
   - ☐ Agree
   - ☐ Strongly Agree
   - ☐ Prefer not to say
9. I am able to explain the concept of pharmacogenomics to patients
   - ☐ Strongly Disagree
   - ☐ Disagree
   - ☐ Neutral

- ☐ Agree
- ☐ Strongly Agree
- ☐ Prefer not to say

10. I am able to counsel patients on their pharmacogenomics test results
    - ☐ Strongly Disagree
    - ☐ Disagree
    - ☐ Neutral
    - ☐ Agree
    - ☐ Strongly Agree
    - ☐ Prefer not to say
11. I am able to explain pharmacogenomics to other healthcare providers
    - ☐ Strongly Disagree
    - ☐ Disagree
    - ☐ Neutral
    - ☐ Agree
    - ☐ Strongly Agree
    - ☐ Prefer not to say

If desired, please elaborate further on your current knowledge of pharmacogenomics: [free text]

Part 3A: Attitudes – Feasibility and Utility

The following 6 questions are regarding your opinions on the feasibility and clinical utility of pharmacogenomics.

1. Pharmacogenomics can enhance medication efficacy
    - ☐ Strongly Disagree
    - ☐ Disagree
    - ☐ Neutral
    - ☐ Agree
    - ☐ Strongly Agree
    - ☐ Prefer not to say
2. Pharmacogenomics can prevent medication adverse drug effects
    - ☐ Strongly Disagree
    - ☐ Disagree
    - ☐ Neutral
    - ☐ Agree
    - ☐ Strongly Agree
    - ☐ Prefer not to say
3. Pharmacogenomic testing is cost-effective
    - ☐ Strongly Disagree
    - ☐ Disagree
    - ☐ Neutral
    - ☐ Agree
    - ☐ Strongly Agree
    - ☐ Prefer not to say
4. I can see myself using pharmacogenomics in my practice setting in the next 10 years
    - ☐ Strongly Disagree
    - ☐ Disagree
    - ☐ Neutral
    - ☐ Agree
    - ☐ Strongly Agree

- ☐ Prefer not to say

5. I want to utilize pharmacogenomics in my practice setting
   - ☐ Strongly Disagree
   - ☐ Disagree
   - ☐ Neutral
   - ☐ Agree
   - ☐ Strongly Agree
   - ☐ Prefer not to say
6. I want to learn more about pharmacogenomics
   - ☐ Strongly Disagree
   - ☐ Disagree
   - ☐ Neutral
   - ☐ Agree
   - ☐ Strongly Agree
   - ☐ Prefer not to say

Part 3B: Attitudes – Education and Implementation

The following 9 questions are regarding your opinions on facilitators and barriers towards pharmacogenomics implementation.

1. If you were to receive further education in pharmacogenomics, which medium would you prefer?
   - ☐ A conference, congress, or meeting
   - ☐ On-site training
   - ☐ A certificate program
   - ☐ Self-study
   - ☐ Small group workshop
   - ☐ Other (please specify other learning mediums for pharmacogenomics education:_)
2. What therapeutic area(s) could be considered for implementation of pharmacogenomics? (select all that apply)
   - ☐ No specialty/ general practice
   - ☐ Emergency
   - ☐ Cardiovascular or Stroke
   - ☐ Psychiatry
   - ☐ Gastroenterology
   - ☐ Infectious Diseases
   - ☐ Oncology
   - ☐ Respiratory
   - ☐ Nephrology
   - ☐ Endocrinology or Diabetes
   - ☐ Geriatrics
   - ☐ Pediatrics
   - ☐ Critical Care or ICU
   - ☐ Palliative Care
   - ☐ Other (please specify: ___________________)
3. What therapeutic area is the <u>best</u> suited for implementation of pharmacogenomics? (select <u>one</u>)
   - ☐ No specialty/ general practice
   - ☐ Emergency
   - ☐ Cardiovascular or Stroke
   - ☐ Psychiatry

- ☐ Gastroenterology
- ☐ Infectious Diseases
- ☐ Oncology
- ☐ Respiratory
- ☐ Nephrology
- ☐ Endocrinology or Diabetes
- ☐ Geriatrics
- ☐ Pediatrics
- ☐ Critical Care or ICU
- ☐ Palliative Care
- ☐ Other (please specify: ___________________)

4. Which profession is <u>best</u> suited to provide patients with initial (pre-test) pharmacogenomic education? (select <u>one</u>)
   - ☐ Physician
   - ☐ Pharmacist
   - ☐ Nurse Practitioner
   - ☐ Registered Nurse
   - ☐ Genetic Counsellor
   - ☐ Other (please specify: ___________)
5. Which profession is <u>best</u> suited to interpret pharmacogenomic test results? (select <u>one</u>)
   - ☐ Physician
   - ☐ Pharmacist
   - ☐ Nurse Practitioner
   - ☐ Registered Nurse
   - ☐ Genetic Counsellor
   - ☐ Other (please specify: ___________)
6. Which profession is <u>best</u> suited to provide patients with post-test pharmacogenomic education and follow-up? (select <u>one</u>)
   - ☐ Physician
   - ☐ Pharmacist
   - ☐ Nurse Practitioner
   - ☐ Registered Nurse
   - ☐ Genetic Counsellor
   - ☐ Other (please specify: ___________)
7. What barriers do you perceive to future pharmacogenomic testing in Alberta hospitals? (select all that apply)
   - ☐ Lack of healthcare provider knowledge
   - ☐ Lack of testing equipment
   - ☐ Cost of testing
   - ☐ Lack of clinical guidelines
   - ☐ Lack of time to provide this service
   - ☐ Transport of test samples
   - ☐ Delay in test results
   - ☐ Ethical concerns
   - ☐ Legal concerns
   - ☐ Social concerns
   - ☐ Lack of patient acceptance
   - ☐ Other (please specify)
8. What is the **most critical** barrier to future pharmacogenomic testing in Alberta hospitals? (select <u>one</u>)
   - ☐ Lack of healthcare provider knowledge

- ☐ Lack of testing equipment
- ☐ Cost of testing
- ☐ Lack of clinical guidelines
- ☐ Lack of time to provide this service
- ☐ Transport of test samples
- ☐ Delay in test results
- ☐ Ethical concerns
- ☐ Legal concerns
- ☐ Social concerns
- ☐ Lack of patient acceptance
- ☐ Other (please specify)

9. What resources do you prefer to access for pharmacogenomic information? (check all that apply)
   - ☐ Pharmacogenomics Knowledge Base (PharmGKB)
   - ☐ Clinical Pharmacogenomics Implementation Consortium (CPIC) guidelines
   - ☐ Dutch Pharmacogenomics Working Group (DPWG) guidelines
   - ☐ Canadian Pharmacogenomics Network for Drug Safety (CPNDS)
   - ☐ Pharmacogene Variation Consortium (PharmVar)
   - ☐ Primary literature (research articles)
   - ☐ LexiComp
   - ☐ Micromedex
   - ☐ Product Monograph / Drug Labels
   - ☐ Other (Please Specify: _____________________________)
   - ☐ I do not know where to look for pharmacogenomic information

If desired, please elaborate further on your opinions and attitudes regarding pharmacogenomics: [free text response]
