# Supplementary material for: Experience, Knowledge, and Perceptions of Pharmacogenomics among Pharmacists and Nurse Practitioners in Alberta Hospitals
Source: Pharmacy (Basel). 2022 Oct 26;10(6):139. doi: 10.3390/pharmacy10060139 (PMC9680290; doi:10.3390/pharmacy10060139)
Supplement: Supplementary file 1 [file pharmacy-10-00139-s001.zip › Table S1 OCT 18 2022.pdf]

Table S1. Demographics of survey respondents by profession. Physicians excluded due to low response rate.

Characteristic	n (%)			
	Pharmacists (n = 91)		Nurse Practitioners (n = 37)	
**Years in Practice***				
Less than 2 years	5	(5.6)	2	(5.4)
2-5 years	13	(14.4)	7	(18.9)
6-10 years	12	(13.3)	14	(37.8)
11-15 years	12	(13.3)	6	(16.2)
16-20 years	13	(14.4)	5	(13.5)
More than 20 years	35	(38.9)	3	(8.1)
**Location of Practice (Number of inhabitants)****				
Rural (0-50,000) or Locum	9	(10.1)	4	(10.8)
Suburban (50,001-250,000)	17	(19.1)	8	(21.6)
Urban (greater than 250,000)	64	(71.9)	25	(67.6)
**Specialty****				
None / general medicine	17	(18.9)	6	(16.2)
Pediatric / neonatal medicine	11	(6.7)	6	(16.2)
Oncology	10	(11.1)	7	(18.9)
Adult intensive care or emergency medicine	10	(11.1)	5	(13.5)
Psychiatry	9	(10.0)	0	(0)
Cardiology and stroke	8	(8.9)	2	(5.4)
Geriatrics	6	(6.7)	3	(8.1)
Infectious diseases	6	(6.7)	1	(2.7)
Pain and palliative care	5	(5.6)	2	(5.4)
Other**	4	(4.4)	3	(8.1)
**Prior Training and Exposure to Pharmacogenomics**				
Prior education	29/84	(34.5)	7/35	(20.0)
Prior use in practice	23/84	(27.4)	5/35	(14.3)

N=134 *two missing responses; **non-cardiac surgery (n=2), human immunodeficiency virus (n=1), neurology (n=1), transplant (n=1), physiatry (n=1).
